# Sudan Virus Persistence in Immune-Privileged Organs of Nonhuman Primate Survivors

**DOI:** 10.3201/eid3102.240983

**Published:** 2025-02

**Authors:** Brittany B. Beavis, Jun Liu, Elizabeth E. Zumbrun, Ashley V. Bryan, April M. Babka, Nancy A. Twenhafel, Derron A. Alves, Margaret L. Pitt, Aysegul Nalca, Xiankun Zeng

**Affiliations:** United States Army Medical Research Institute of Infectious Diseases, Fort Detrick, Frederick, Maryland, USA (B.B. Beavis, J. Liu, E.E. Zumbrun, A.V. Bryan, A.M. Babka, N.A. Twenhafel, D.A. Alves, M.L. Pitt, A. Nalca, X. Zeng); Chenega Corporation, Chesapeake, Virginia, USA (J. Liu)

**Keywords:** Sudan virus, SUDV, Ebola, Ebola virus, filovirus, Ebola disease, EBOD, Ebola virus disease, EVD, virus persistence, persistent infection, immune-privileged organ, viruses, nonhuman primates

## Abstract

After the 2022–2023 Sudan virus (SUDV) disease outbreak in Uganda, we studied SUDV persistence in nonhuman primates that had survived acute infection without therapeutic intervention. We identified SUDV persistence in the vitreous chamber and immediately adjacent tissue in the eyes as well as in the seminiferous tubules in the testes but not in common target organs typically infected during the acute phase of disease. Specifically, SUDV persists primarily in macrophages in the eyes and Sertoli cells in the testes. Ocular and testicular SUDV persistence in nonhuman primates is accompanied by tissue damage, including inflammatory cell invasion. Our study suggests that long-term follow-up efforts are needed to reduce possible recrudescent disease and reignition of outbreaks caused by virus persistence in human survivors of SUDV infection.

Members of the viral genera *Orthoebolavirus* and *Orthomarburgvirus* in the family *Filoviridae* cause severe viral hemorrhagic fevers in humans; case-fatality rates are high. Ebola disease is caused by infection with Bundibugyo virus, Ebola virus (EBOV), Sudan virus (SUDV), or Taï Forest virus, whereas Marburg disease is caused by infection with Marburg virus (MARV) or Ravn virus (*1*,*2*). Most Ebola disease outbreaks in humans to date have been caused by EBOV and SUDV. Before the 2013‒2016 epidemic of Ebola virus disease (EVD, caused by EBOV only) in West Africa and the 2018‒2020 EVD outbreak in the Democratic Republic of the Congo, the largest Ebola disease outbreak was in the Gulu District of Uganda, caused by SUDV; 425 persons were infected and 224 deaths were recorded (*3*,*4*). SUDV has been responsible for the second most cases of filovirus disease, after EBOV; 8 sporadic SUDV outbreaks have been reported in equatorial Africa since discovery of the virus in 1976 (*5*). The recent outbreak caused by SUDV in Uganda (September 2022–January 2023) resulted in 142 confirmed cases and 55 deaths (*6*). Although substantial progress has been made in preclinical studies of vaccines and therapeutics against SUDV, no approved vaccines or therapeutics against SUDV are available for patients. Although therapeutics and vaccines licensed for use against EBOV are available, EBOV is antigenically distinct, and current evidence suggests that those products would be ineffective against SUDV (*7*).

Persistent filovirus infection was originally identified in 1967, during the first Marburg disease outbreak in Marburg, Germany (*8*). In that case, MARV persisted in the seminal fluid of a convalescent patient, resulting in sexual transmission of MARV to his wife about 2 months after his recovery. Before the 2013‒2016 epidemic of EVD in western Africa, filovirus persistence in the eyes and semen of convalescent survivors was sparsely reported (*9*,*10*). The studies performed among unprecedented numbers of EVD survivors of that epidemic demonstrated a previously underappreciated and unfortunate fact of EBOV infection: the persistence of EBOV in immune-privileged organs, associated body fluids, or both, including brain/cerebrospinal fluid, eyes/ocular fluid, and testes/seminal fluids. Among patients who survive acute EVD, the virus remains in those tissues despite virus clearance from blood, EBOV-specific immune responses, and apparent clinical recovery. Multiple disease flare-ups or re-emergence events associated with virus persistence were reported and attributed to sexual transmission or breastfeeding during the 2013‒2016 EVD epidemic (*11*). Persistent infectious virus has been isolated from ocular fluid of an EVD survivor with recrudescent uveitis and from the cerebrospinal fluid of an EVD survivor with meningoencephalitis (*12*,*13*). At the end of the 2018‒2020 EVD outbreak in the Democratic Republic of the Congo, a lethal relapse in a survivor 6 months after treatment with monoclonal antibody mAb114 led to multiple subsequent transmission events and extended the outbreak and response efforts another 6 months (*14*,*15*). More recently, genetic epidemiology suggested that 3 separate EVD outbreaks (in the Democratic Republic of the Congo during February 2021 and October 2021 and in Guinea during February 2021) were probably associated with virus persistence in a survivor of a prior local outbreak rather than with spillover from an unknown zoonotic source. In particular, the 2021 Guinea EVD outbreak reemerged from a persistently infected survivor of the major 2013–2016 EVD epidemic (at least 5 years earlier) (*16*). Those events have resulted in a paradigm shift with regard to knowledge of EBOV persistence and outbreak response and prevention.

Studies of EBOV persistence in humans and experimentally infected nonhuman primates (NHPs) have revolutionized our knowledge of EBOV infection and changed the guidelines of clinical operation and the recommendations of the World Health Organization for EVD survivors. MARV persistence in NHP survivors has also been investigated (*17*). However, our knowledge of SUDV persistence has lagged substantially, although SUDV has been responsible for numerous filovirus outbreaks. With this study, we sought to fill the knowledge gap and identify and characterize ocular and testicular SUDV persistence in NHPs that naturally survived experimental SUDV exposure without therapeutic interventions. 

## Materials and Methods

### Study Design

We searched the internal pathology database at United States Army Medical Research Institute of Infectious Diseases (USAMRIID) for NHPs that had survived >28 days after exposure to SUDV without therapeutic interference (defined as administration of experimental therapeutics or vaccines). Meeting the inclusion criteria were 8 rhesus monkeys (*Macaca mulatta*, also known as rhesus macaques), 5 crab-eating macaques (*Macaca fascicularis*, also known as cynomologus macaques), and 3 vervets (*Chlorocebus aethiops sabaeus*, also known as African green monkeys). We selected the immune-privileged organs (eye, brain, and testes) and common target organs (liver, spleen, lymph nodes) of those NHPs for analysis. From the USAMRIID Pathology Division tissue archives, we retrieved formalin-fixed paraffin-embedded (FFPE) tissue samples from those animals. We verified detection of SUDV infection by >2 different methods (in situ hybridization, immunohistochemistry, immunofluorescence staining).

We conducted our research under an Institutional Animal Care and Use Committee– approved protocol in compliance with the Animal Welfare Act, Public Health Service policy, and other federal statutes and regulations relating to animals and experiments involving animals. The facility where our research was conducted is accredited by the Association for Assessment and Accreditation of Laboratory Animal Care International and adheres to principles stated in the Guide for the Care and Use of Laboratory Animals, National Research Council, 2011.

### Animals

We experimentally exposed the 16 NHPs to various doses of the Boniface, Gulu, or Yambio variants of SUDV via aerosol or intramuscular route (Appendix Table). For histologic evaluation, we processed tissue sections in accordance with routine hematoxylin and eosin staining procedures and used sections of tissues from an uninfected rhesus macaque and an uninfected crab-eating macaque as controls.

### RNA In Situ Hybridization

To detect SUDV genomic RNA in FFPE tissues, we performed RNA in situ hybridization (ISH) by using an RNAscope 2.5 HD Detection (RED) kit (Advanced Cell Diagnostics, https://acdbio.com), according to the manufacturer’s instructions. In brief, Advanced Cell Diagnostics designed and synthesized an ISH probe targeting the genomic fragment of the SUDV nucleoprotein (NP) gene. We used uninfected NHP tissue sections as negative controls and SUDV-infected NHP tissue sections as positive controls. Tissue sections deparaffinized with Xyless II (Val Tech Diagnostics, https://valtechnologies.com) underwent a series of ethanol washes and peroxidase blocking, were heated in kit-provided decrosslinking buffer, and were then digested by kit-provided proteinase. We exposed sections to ISH target probe pairs and incubated them at 40°C in a hybridization oven for 2 hours. After rinsing the sections, we amplified the ISH signal by using kit-provided preamplifier and amplifier conjugated to alkaline phosphatase and incubated them with a Fast Red substrate solution for 10 minutes at room temperature. We then stained the sections with hematoxylin, air-dried them, and placed coverslips.

### Immunohistochemistry

We performed SUDV immunohistochemistry testing on FFPE tissue sections by using the EnVision Detection System (Dako Agilent Pathology Solutions, https://www.agilent.com). We used uninfected NHP tissue sections as negative controls and SUDV-infected NHP tissue sections as positive controls. After we deparaffinized, rehydrated, and blocked the sections with methanol/hydrogen peroxide, we stained the slides by using rabbit polyclonal anti-SUDV VP40 antibody (IBT Bioservices, https://ibtbioservices.com) at a dilution of 1:4,000, followed by a horseradish peroxidase-conjugated secondary anti-mouse polymer (Dako Agilent Pathology Solutions). We exposed all slides to brown chromogenic substrate, 3,3′-diaminobenzidine (Dako Agilent Pathology Solutions), counterstained them with hematoxylin, dehydrated them, and placed coverslips.

### Immunofluorescence Staining

We deparaffinized FFPE tissue sections by using Xyless II (Val Tech Diagnostics) and a series of ethanol washes. To reverse formaldehyde crosslinks, we heated the sections in Tris-EDTA buffer (10 mM Tris base, 1 mM EDTA solution, 0.05% Tween 20, pH 9.0) for 20 minutes. After rinses with phosphate-buffered saline (PBS), we blocked pH 7.4 sections with CAS-Block ((Thermo Fisher Scientific, https://www.thermofisher.com) containing 5% normal goat serum (Millipore Sigma, https://www.sigmaaldrich.com) for 1 hour at room temperature or overnight at 4°C. We then incubated sections with the primary antibodies overnight at 4°C or for 2 hours at room temperature as follows: a rabbit polyclonal antibody against SUDV VP40 antibody (IBT Bioservices) at a dilution of 1:500, a mouse monoclonal anti-human CD68 antibody (Dako Agilent Pathology Solutions) at a dilution of 1:200, a rabbit polyclonal anti-CD3 antibody at a dilution of 1:200 (Dako Agilent Pathology Solutions), a rabbit polyclonal anti-CD4 antibody (Abcam, https://www.abcam.com) or a mouse monoclonal anti-CD8α antibody at a dilution of 1:200 (Thermo Fisher Scientific). After rinsing sections in PBS + 0.1% Tween-20, we incubated them with secondary goat Alexa Fluor 488-conjugated anti-rabbit antibody and with goat Alexa Fluor 561 anti-mouse antibody (Thermo Fisher Scientific) for 1 hour at room temperature. We counterstained sections with 4′,6-diamidino-2-phenylindole and placed coverslips by using fluorescent mounting media (Dako Agilent Pathology Solutions). Images were captured on an LSM 880 confocal microscope (Zeiss, https://www.zeiss.com) and processed by using open-source ImageJ software (National Institutes of Health, Bethesda, MD, USA).

## Results

### SUDV Virus Persistence 

 Of the 16 survivors, 4 (25%) had detectable SUDV genomic RNA in eye or testis tissues. Specifically, 3 (23.1%) of 13 NHPs (no eye and brain tissues were collected for 3 surviving NHPs; Appendix Table) had SUDV genomic RNA in eye tissues and 1 (9%) of 11 NHPs (the other 5 were female) had SUDV genomic RNA in testis tissues. In contrast, SUDV genomic RNA was undetectable in the brain tissues of all 13 NHPs for which brain tissues were collected, the ovary tissues of all 5 female survivors, and common acute SUDV infection target organ tissues (liver, lymph node, and spleen) of all 16 NHP survivors (Appendix Table).

### Ocular Sudan Virus Persistence 

We previously reported ocular EBOV persistence in NHPs that survived EBOV exposure with or without therapeutic interventions (*18*,*19*). Similarly, in this study, using ISH we detected SUDV genomic RNA in the vitreous chamber and the interface between the vitreous chamber and its adjacent structures within the eyes of 3 (23.1%) of 13 NHP survivors (Appendix Figure 1, panels A–N). In contrast, we did not detect genomic SUDV RNA in brain, liver, lymph node, spleen, or testicular tissues from the same 3 NHPs (Appendix Table). To identify the cellular targets of persistent SUDV infection, we stained survivor eye tissues by using immunofluorescence and an SUDV NP antibody and antibody against a macrophage marker, CD68. SUDV NPs were detected primarily in CD68+ macrophages (Appendix Figure 1, panels O–P), suggesting that SUDV primarily persists in ocular macrophages.

### Uveitis, Retinitis, and Vitritis 

A high prevalence of ophthalmic sequelae, including sight-threatening uveitis, in EVD survivors has been reported (*20*,*21*). To examine the ocular complications in SUDV NHP survivors, we performed histopathologic evaluation of all eye tissues. Overall, 7 (53.8%) of 13 NHP survivors, including the 3 survivors with ocular SUDV persistence, displayed unilateral or bilateral inflammation of mild to moderate severity in multiple locations. Uveitis, characterized by lymphoplasmacytic infiltration in ciliary body, choroid, and iris, was observed in 7 (100%) of 7 survivors with ocular lesions. Unilateral or bilateral retinitis, characterized by multifocal perivascular accumulation of mononuclear cells and stromal infiltrates of plasma cells, was also observed in the same NHPs. Vitritis, unilateral or bilateral, characterized by infiltration of plasma cells, macrophages, and lymphocytes in the vitreous chamber adjacent to ciliary body, lens, and retina was observed in 6 (85.7%) of 7 of NHP survivors with ocular lesions (Appendix Table, Figure 2, panels A–C, E–G). Optic neuritis was observed in 5 (71.4%) of 7 survivors with ocular lesions, and optic perineuritis was observed in 4 (57.1%) of 7 survivors with ocular lesions (Appendix Table, Figure 2, panels D, H). Less commonly, we detected conjunctivitis, keratitis, and scleritis. Immunofluorescence staining further characterized the infiltrating cells as predominantly CD3+ T cells and CD68+ macrophages in the uvea, retina, vitreous chamber, and optic nerve (Appendix Figure 2, panels I–P). Of note, most T cells in those sites are CD8+ cytotoxic cells rather than CD4+ helper T cells (Appendix Figure 2, panels Q–T). Our data suggest that ocular lesions persist in a subset of NHPs that survive acute SUDV infection.

### Testicular SUDV Persistence 

One (9%) of 11 NHP survivors had detectable SUDV genomic RNA (Appendix Figure 3, panels A–B) in testicular tissue. SUDV antigen was also detected at the same location via immunohistochemistry (Appendix Figure 3, panel C). Of note, both SUDV genomic RNA and SUDV antigen were specifically detected in the seminiferous tubules, sites of immune privilege and sperm production (Appendix Figure 3, panels B–C). Immunofluorescence staining confirmed SUDV antigen presence in the seminiferous tubules, specifically within testicular Sertoli cells. The presence of virus antigen and genomic RNA after resolution of the clinical course of disease suggests a persistent state of infection at that location. We performed histologic analyses of testicular tissues from 11 NHP survivors with available tissues. The NHP with detectable SUDV genomic RNA and antigen demonstrated multifocal interstitial orchitis characterized by expansion of the interstitium by a lymphoplasmacytic infiltrate (Appendix Figure 3, panels F–G). Seminiferous tubules in those areas demonstrated degeneration, characterized by vacuolation, loss of Sertoli cells, and a lack of organized spermatogenesis.

Consistent with histologic analysis, immunofluorescence staining demonstrated that CD68+ macrophages, CD3+ T cells, and CD20+ B cells infiltrated interstitial tissues and seminiferous tubules of testicular sites with SUDV persistence (Appendix Figure 3, panels H–K). We detected abundant IgG in the interstitial tissues and seminiferous tubules but not in uninfected control testicular tissues (Appendix Figure 3, panels J–K). However, whether the IgG responses are SUDV antigen–specific remains to be investigated. Our data suggest that persistent testicular SUDV infection results in orchitis and loss or partial loss of immune privilege.

## Discussion

Asymptomatic persistent infection in clinically recovered Ebola patients may cause recrudescent disease and may spark new outbreaks months, or even years, later. The 2022–2023 outbreak of Ebola disease caused by SUDV in Uganda reminded the field of the need for more information about the pathogenesis and transmission of SUDV. Our data demonstrate that SUDV persists beyond the conclusion of acute clinical disease, specifically in the vitreous chamber and its adjacent structures of the eyes and in the seminiferous tubules of the testes in some NHPs that naturally survived experimental exposure of SUDV without therapeutic treatment. Our data suggest that persistence is linked to the presence of ongoing inflammatory infiltrates. The primary targets of SUDV persistence are macrophages in the eyes and Sertoli cells in the testes.

Consistent with our finding of ocular SUDV persistence and inflammation, a previous animal efficacy study briefly mentioned inflammatory ocular lesions, including uveitis, and detectable viral RNA in the eye tissues of NHPs that survived experimental SUDV exposure after combination therapy with remdesivir and monoclonal antibodies or after monotherapy with remdesivir or monoclonal antibodies (*22*). Natural history studies demonstrate that SUDV infection in NHPs, including rhesus monkeys and crab-eating macaques, results in systemic viremia and characteristic clinical signs, recapitulating many manifestations of human SUDV disease (*23*–*27*). Although SUDV persistence and related sequelae have not been reported for human survivors, given our NHP data and similar pathogenesis between SUDV and EBOV, we posit that SUDV can most likely persist in immune-privileged organs in patients. The clinical observations and data of the Survivor Care Program, established by public health authorities during the 2022–2023 SUDV disease outbreak in Uganda but not yet published, may help prove such deduction (*28*).

SUDV persistence was detected only in the eye and testicular tissues but not in the brain tissues we analyzed. We suspect that SUDV could persist in the brain of NHP survivors because we previously reported virus persistence in the brain of NHPs exposed to EBOV (*19*). However, in that study we identified that NHP survivors experiencing EBOV persistence in the brain had higher viral loads in the blood than NHP survivors without EBOV persistence in the brain. The lack of brain persistence in those NHP survivors might result from lower viral load, and NHPs with the high viral load needed to result in brain SUDV persistence might have succumbed during the acute phase of disease.

Among its limitations, the retrospective nature of our study means that no virologic data are available to determine infectious virus in the eye and testicular tissues. Second, our observations of SUDV persistence were based on NHP survivors at ≈30 days after exposure. Analysis of NHPs at different stages of the convalescent disease course could provide information about the dynamics of virus persistence. Last, all NHP survivors that we report are natural survivors without therapeutic interference. SUDV persistence should be further investigated in future NHP studies evaluating efficacy of medical countermeasures candidates, including monoclonal antibodies, small molecules, and vaccines. Of note, a recent study demonstrated that infectious SUDV was undetectable in the immune-privileged tissues of NHPs that survived SUDV exposure after treatment with obeldesivir, an oral alternative to parenterally administered remdesivir (*29*).

Persistent SUDV infection in even a very small subset of individual human survivors has consequences for the individuals and for public health, particularly with respect to the potential for reignition of human-to-human transmission chains leading to a new outbreak. Our study suggests the need for long-term follow-up (clinical and potentially virologic surveillance) of convalescent SUDV patients to prevent disease recrudescence and reignition of outbreaks.

Appendix Additional information for study of Sudan virus persistence in immune-privileged organs of nonhuman primates.

## References

[1] Jacob ST, Crozier I, Fischer WA II, Hewlett A, Kraft CS, Vega MA, et al. Ebola virus disease. Nat Rev Dis Primers. 2020;6:13. 10.1038/s41572-020-0147-3 32080199 PMC7223853

[2] Biedenkopf N, Bukreyev A, Chandran K, Di Paola N, Formenty PBH, Griffiths A, et al. Renaming of genera *Ebolavirus* and *Marburgvirus* to *Orthoebolavirus* and *Orthomarburgvirus*, respectively, and introduction of binomial species names within family *Filoviridae.* Arch Virol. 2023;168:220. 10.1007/s00705-023-05834-2 37537381

[3] Centers for Disease Control and Prevention. Ebola virus disease outbreak history [cited 2024 May 28]. https://wwwcdcgov/ebola/outbreaks/index.html

[4] Towner JS, Rollin PE, Bausch DG, Sanchez A, Crary SM, Vincent M, et al. Rapid diagnosis of Ebola hemorrhagic fever by reverse transcription-PCR in an outbreak setting and assessment of patient viral load as a predictor of outcome. J Virol. 2004;78:4330–41. 10.1128/JVI.78.8.4330-4341.2004 15047846 PMC374287

[5] Languon S, Quaye O. Filovirus disease outbreaks: a chronological overview. Virology (Auckl). 2019;10:1178122X19849927. 10.1177/1178122X19849927 PMC6589952 31258326

[6] Balinandi S, Whitmer S, Mulei S, Nassuna C, Pimundu G, Muyigi T, et al. Molecular characterization of the 2022 Sudan virus disease outbreak in Uganda. J Virol. 2023;97:e0059023. 10.1128/jvi.00590-23 37750724 PMC10617429

[7] Ibrahim SK, Ndwandwe DE, Thomas K, Sigfrid L, Norton A. Sudan virus disease outbreak in Uganda: urgent research gaps. BMJ Glob Health. 2022;7:e010982. 10.1136/bmjgh-2022-010982 36585031 PMC9809242

[8] Martini GA, Schmidt HA. Spermatogene Ubertragung des “Virus Marburg”. (Erreger der “Marburger Affenkrankheit”). Klin Wochenschr. 1968;46:398–400. 10.1007/BF01734141 4971902

[9] Schindell BG, Webb AL, Kindrachuk J. Persistence and sexual transmission of filoviruses. Viruses. 2018;10:683. 10.3390/v10120683 30513823 PMC6316729

[10] Di Paola N, Sanchez-Lockhart M, Zeng X, Kuhn JH, Palacios G. Viral genomics in Ebola virus research. Nat Rev Microbiol. 2020;18:365–78. 10.1038/s41579-020-0354-7 32367066 PMC7223634

[11] Subissi L, Keita M, Mesfin S, Rezza G, Diallo B, Van Gucht S, et al. Ebola virus transmission caused by persistently infected survivors of the 2014–2016 outbreak in West Africa. J Infect Dis. 2018;218(suppl_5):S287–91. 10.1093/infdis/jiy280 29920602 PMC6249578

[12] Varkey JB, Shantha JG, Crozier I, Kraft CS, Lyon GM, Mehta AK, et al. Persistence of Ebola virus in ocular fluid during convalescence. N Engl J Med. 2015;372:2423–7. 10.1056/NEJMoa1500306 25950269 PMC4547451

[13] Jacobs M, Rodger A, Bell DJ, Bhagani S, Cropley I, Filipe A, et al. Late Ebola virus relapse causing meningoencephalitis: a case report. Lancet. 2016;388:498–503. 10.1016/S0140-6736(16)30386-5 27209148 PMC4967715

[14] Mbala-Kingebeni P, Pratt C, Mutafali-Ruffin M, Pauthner MG, Bile F, Nkuba-Ndaye A, et al. Ebola virus transmission initiated by relapse of systemic Ebola virus disease. N Engl J Med. 2021;384:1240–7. 10.1056/NEJMoa2024670 33789012 PMC7888312

[15] Crozier I, Britson KA, Wolfe DN, Klena JD, Hensley LE, Lee JS, et al. The evolution of medical countermeasures for Ebola virus disease: lessons learned and next steps. Vaccines (Basel). 2022;10:1213. 10.3390/vaccines10081213 36016101 PMC9415766

[16] Keita AK, Koundouno FR, Faye M, Düx A, Hinzmann J, Diallo H, et al. Resurgence of Ebola virus in 2021 in Guinea suggests a new paradigm for outbreaks. Nature. 2021;597:539–43. 10.1038/s41586-021-03901-9 34526718

[17] Coffin KM, Liu J, Warren TK, Blancett CD, Kuehl KA, Nichols DK, et al. Persistent Marburg virus infection in the testes of nonhuman primate survivors. Cell Host Microbe. 2018;24:405–416.e3. 10.1016/j.chom.2018.08.003 30173956

[18] Zeng X, Blancett CD, Koistinen KA, Schellhase CW, Bearss JJ, Radoshitzky SR, et al. Identification and pathological characterization of persistent asymptomatic Ebola virus infection in rhesus monkeys. Nat Microbiol. 2017;2:17113. 10.1038/nmicrobiol.2017.113 28715405

[19] Liu J, Trefry JC, Babka AM, Schellhase CW, Coffin KM, Williams JA, et al. Ebola virus persistence and disease recrudescence in the brains of antibody-treated nonhuman primate survivors. Sci Transl Med. 2022;14:eabi5229. 10.1126/scitranslmed.abi5229 35138912

[20] Sneller MC, Reilly C, Badio M, Bishop RJ, Eghrari AO, Moses SJ, et al.; PREVAIL III Study Group. A longitudinal study of Ebola sequelae in Liberia. N Engl J Med. 2019;380:924–34. 10.1056/NEJMoa1805435 30855742 PMC6478393

[21] Shantha JG, Mattia JG, Goba A, Barnes KG, Ebrahim FK, Kraft CS, et al. Ebola Virus Persistence in Ocular Tissues and Fluids (EVICT) Study: reverse transcription-polymerase chain reaction and cataract surgery outcomes of Ebola survivors in Sierra Leone. EBioMedicine. 2018;30:217–24. 10.1016/j.ebiom.2018.03.020 29622497 PMC5952345

[22] Cross RW, Bornholdt ZA, Prasad AN, Woolsey C, Borisevich V, Agans KN, et al. Combination therapy with remdesivir and monoclonal antibodies protects nonhuman primates against advanced Sudan virus disease. JCI Insight. 2022;7:e159090. 10.1172/jci.insight.159090 35413016 PMC9220838

[23] Zumbrun EE, Bloomfield HA, Dye JM, Hunter TC, Dabisch PA, Garza NL, et al. A characterization of aerosolized Sudan virus infection in African green monkeys, cynomolgus macaques, and rhesus macaques. Viruses. 2012;4:2115–36. 10.3390/v4102115 23202456 PMC3497044

[24] Carbonnelle C, Moroso M, Pannetier D, Godard S, Mély S, Thomas D, et al. Natural history of *Sudan ebolavirus* to support medical countermeasure development. Vaccines (Basel). 2022;10:963. 10.3390/vaccines10060963 35746571 PMC9228702

[25] Woolsey C, Fears AC, Borisevich V, Agans KN, Dobias NS, Prasad AN, et al. Natural history of *Sudan ebolavirus* infection in rhesus and cynomolgus macaques. Emerg Microbes Infect. 2022;11:1635–46. 10.1080/22221751.2022.2086072 35657325 PMC9225728

[26] Marzi A, Fletcher P, Feldmann F, Saturday G, Hanley PW, Feldmann H. Species-specific immunogenicity and protective efficacy of a vesicular stomatitis virus-based Sudan virus vaccine: a challenge study in macaques. Lancet Microbe. 2023;4:e171–8. 10.1016/S2666-5247(23)00001-0 36739878 PMC10010116

[27] Alfson KJ, Goez-Gazi Y, Gazi M, Chou YL, Niemuth NA, Mattix ME, et al. Development of a well-characterized cynomolgus macaque model of Sudan virus disease for support of product development. Vaccines (Basel). 2022;10:1723. 10.3390/vaccines10101723 36298588 PMC9611481

[28] World Health Organization. Ebola disease caused by Sudan ebolavirus—Uganda [cited 2024 May 28]. https://wwwwhoint/emergencies/disease-outbreak-news/item/2023-DON433

[29] Cross RW, Woolsey C, Chu VC, Babusis D, Bannister R, Vermillion MS, et al. Oral administration of obeldesivir protects nonhuman primates against *Sudan ebolavirus.* Science. 2024;383:eadk6176. 10.1126/science.adk6176 38484056

