# Supplementary material for: Sudan Virus Persistence in Immune-Privileged Organs of Nonhuman Primate Survivors
Source: Emerg Infect Dis. 2025 Feb;31(2):275–80. doi: 10.3201/eid3102.240983 (PMC11845127; doi:10.3201/eid3102.240983)
Supplement: Appendix — Additional information for study of Sudan virus persistence in immune-privileged organs of nonhuman primates. [file 24-0983-Techapp-s1.pdf]

Article DOI: https://doi.org/10.3201/eid3102.240983

EID cannot ensure accessibility for supplementary materials supplied by authors. Readers who have difficulty accessing supplementary content should contact the authors for assistance.

Sudan Virus Persistence in Immune-Privileged Organs of Nonhuman Primates

Appendix

Appendix Table. Information on 16 nonhuman primate survivors screened for Sudan virus persistence

NHP no., species, sex, age (y)	Days PE	Variant, Target challenging dose, and route	Result of SUDV ISH							Histopathology			
			Liver	Spleen	L.N.	Testis/ Ovary	Left eye	Right eye	Brain	Testis	Left eye	Right eye	Brain
1, R, F	30	Boniface, 1 PFU, AE	–	–	–	–	–	–	–	NA	WNL	Uveitis***, retinitis***, vitritis**, optic neuritis*, keratitis*	WNL
2, V, F	30	Boniface, 1 PFU, AE	–	–	–	–	–	–	–	NA	WNL	WNL	WNL
3, R, M	29	Boniface, 1 PFU, AE	–	–	–	–	–	–	–	WNL	Uveitis**, retinitis**, vitritis*, optic neuritis**, optic perineuritis*, scleritis*, keratitis*	WNL	WNL
4, R, M	28	Boniface, 5 PFU, AE	–	–	–	–	+	+	–	WNL	Uveitis**, retinitis**, vitritis*, optic perineuritis*, keratitis*, scleritis*, conjunctivitis*	Uveitis**, retinitis**, vitritis**, optic perineuritis*, optic neuritis*, scleritis*	WNL
5, V, F	28	Boniface, 5 PFU, AE	–	–	–	–	–	–	–	NA	Uveitis**, retinitis*, vitritis*, optic perineuritis*	WNL	WNL
6, V, M	28	Boniface, 10 PFU, AE	–	–	–	–	NA	NA	NA	WNL	NA	NA	NA
7, R, M	28	Boniface, 10 PFU, AE	–	–	–	–	NA	NA	NA	WNL	NA	NA	NA

NHP no., species, sex, age (y)	Days PE	Variant, Target challenging dose, and route	Result of SUDV ISH							Histopathology			
			Liver	Spleen	L.N.	Testis/ Ovary	Left eye	Right eye	Brain	Testis	Left eye	Right eye	Brain
8, C, M	28	Boniface, 10 PFU, AE	–	–	–	–	NA	NA	NA	WNL	NA	NA	NA
9, R, F, 3.8#	28	Boniface, 50 PFU, AE	–	–	–	–	+	–	–	NA	Uveitis**, retinitis*, vitritis**, optic perineuritis*, conjunctivitis**	Uveitis**, retinitis**, vitritis*, optic perineuritis*, optic neuritis*, scleritis*	WNL
10, R, M, 4.8	37	Boniface, 1,000 PFU, AE	–	–	–	–	–	–	–	WNL	WNL	WNL	WNL
11, R, F,3.4	37	Boniface, 1,000 PFU, AE	–	–	–	–	–	–	–	NA	WNL	WNL	WNL
12, R, M, 4.1	37	Boniface,1,000 PFU, AE	–	–	–	–	–	–	–	WNL	WNL	Uveitis*, optic neuritis*, retinitis*	WNL
13, C, M, 4.8	28	Gulu, 1,000 PFU, IM	–	–	–	+	–	–	–	Orchitis	WNL	Uveitis**, retinitis**, vitritis*	WNL
14, C, M, 5.8	35	Yambio, 1,000 PFU, IM	–	–	–	–	–	+	–	WNL	WNL	WNL	WNL
15, C, M, 5.8	30	Gulu, 1,000 PFU, IM	–	–	–	–	–	–	–	WNL	WNL	WNL	WNL
16, C, M, 5.3	28	Gulu, 1,000 PFU, IM	–	–	–	–	–	–	–	WNL	WNL	WNL	WNL

R, rhesus monkey; V, vervet; C, crab-eating macaque; PE, post-exposure; PFU, plaque-forming units; LN, lymph node; –, ISH-negative; +, ISH-positive; AE, aerosol exposure; IM, intramuscular injection; NA, not available; WNL, within normal limits; * minimal; **, mild to moderate; #, this animal was previously reported (*1*).

A

% Survivors with ocular SUDV persistence	% Survivors with uveitis and other eye inflammation
23.1% (3 of 13)	53.8% (7 of 13)

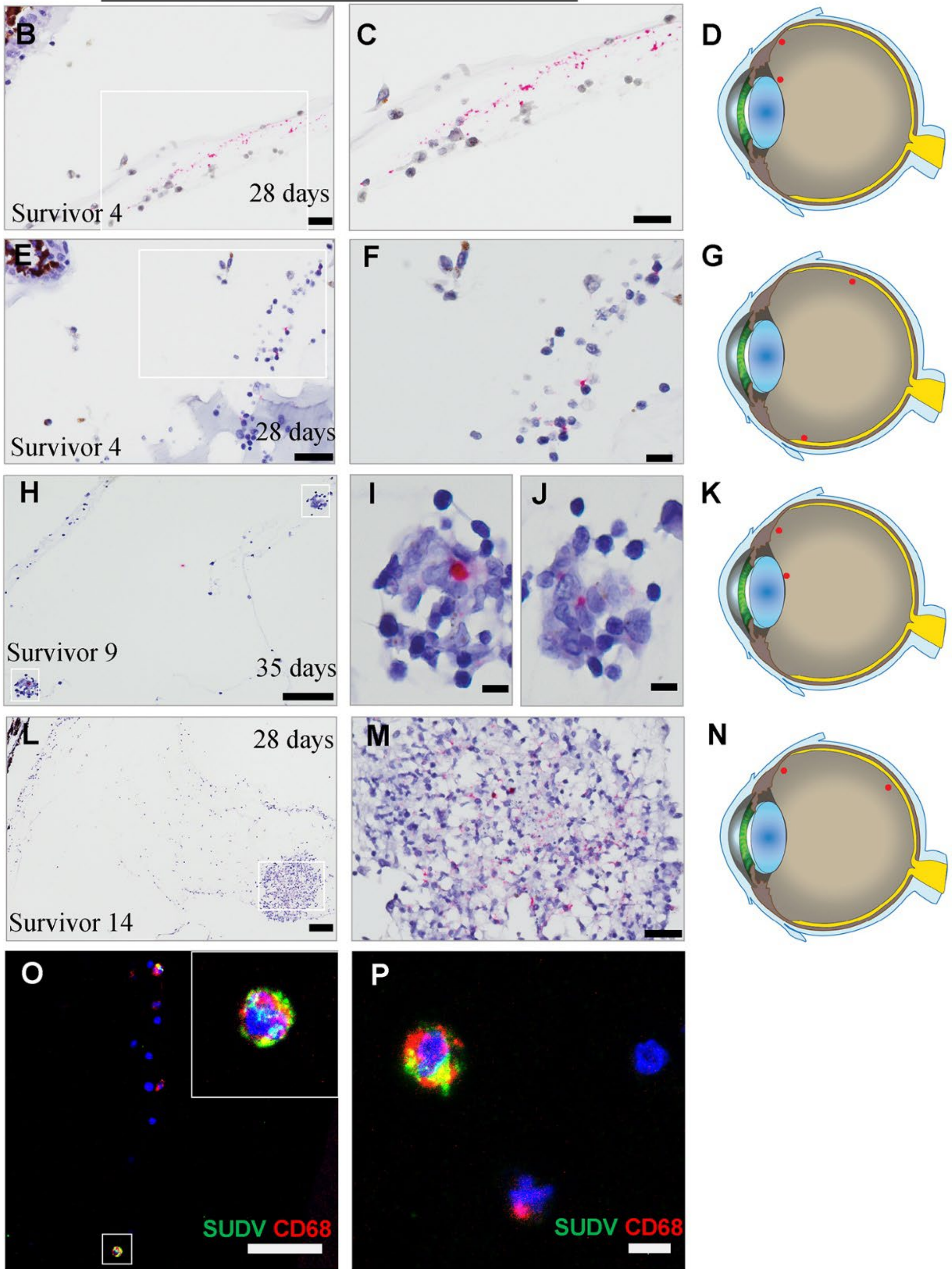

Appendix Figure 1. Sudan virus persistence in the eyes of 3 (23.1%) of 13 nonhuman primates (NHPs) that had survived experimental acute infection without therapeutic intervention and uveitis and other inflammation in the eyes of 7 (53.8%) NHP survivors. B–N) SUDV genomic RNA (red) was detected in the cells in the vitreous chamber, or at the interface between vitreous chamber and its adjacent structures,

including ciliary bodies, lens, and retina in NHP survivors 4, 9, and 14 by using RNA in situ hybridization (ISH). C, F, I, J, and M are insets of B, E, H, and L at high magnification, respectively. D, G, K, and N are schematic representations of SUDV-infected regions (red dots) in the eye. Nuclei were counterstained blue with hematoxylin. O–P) Immunofluorescence staining demonstrates SUDV antigen (green) was detected in CD68⁺ macrophages (red, arrows) in the eyes of survivor 4 and survivor 9. O) Inset at the top right shows the area of white box at bottom at a high magnification. Scale bars indicates 20 μm (B–C, E–F, I–J, and M); 100 μm (H); 200 μm (L), 50 μm (O), and 5 μm (P).

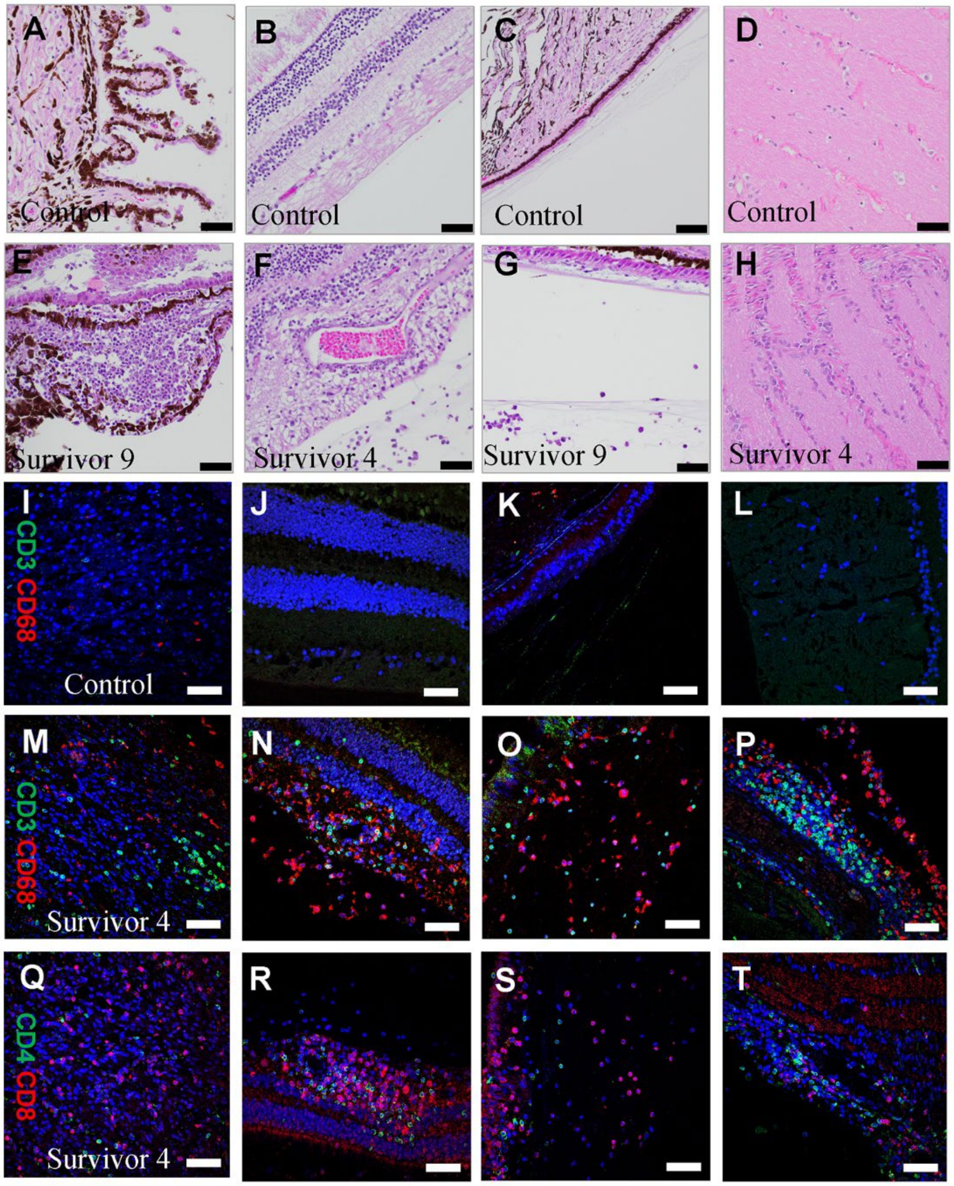

Appendix Figure 2. Uveitis, retinitis, vitritis, and optic neuritis associated with ocular Sudan virus (SUDV) persistence in nonhuman primates (NHPs) after experimental acute infection without therapeutic intervention. A–H) Eye tissue sections of an uninfected control NHP and survivors 4 and 9 stained with

hematoxylin and eosin (H&E). In comparison with the normal histologic structure of ciliary body/process (A), retina (B), vitreous body (B–C), and optical nerve (D), histopathologic evidence of uveitis (E), retinitis (F), vitritis (G), and optic neuritis (H) in NHP survivors with ocular SUDV persistence. I–P) Immunofluorescence staining demonstrating infiltration of $CD3^+$ T cells (green) and $CD68^+$ macrophages (red) in eye tissues with SUDV persistence (M–P), compared with uninfected control eye tissues (I–L). Q–T) Immunofluorescence staining demonstrating that most T cells are $CD8^+$ cytotoxic T cells (red) and a small portion of T cells are $CD4^+$ helper T cells in the eye tissues with SUDV persistence. Nuclei were counterstained blue with 4′,6-diamidino-2-phenylindole. Scale bars indicate 50 μm.

A

% Survivors with testicular SUDV persistence	% Survivors with orchitis
9% (1 of 11)	9% (1 of 11)

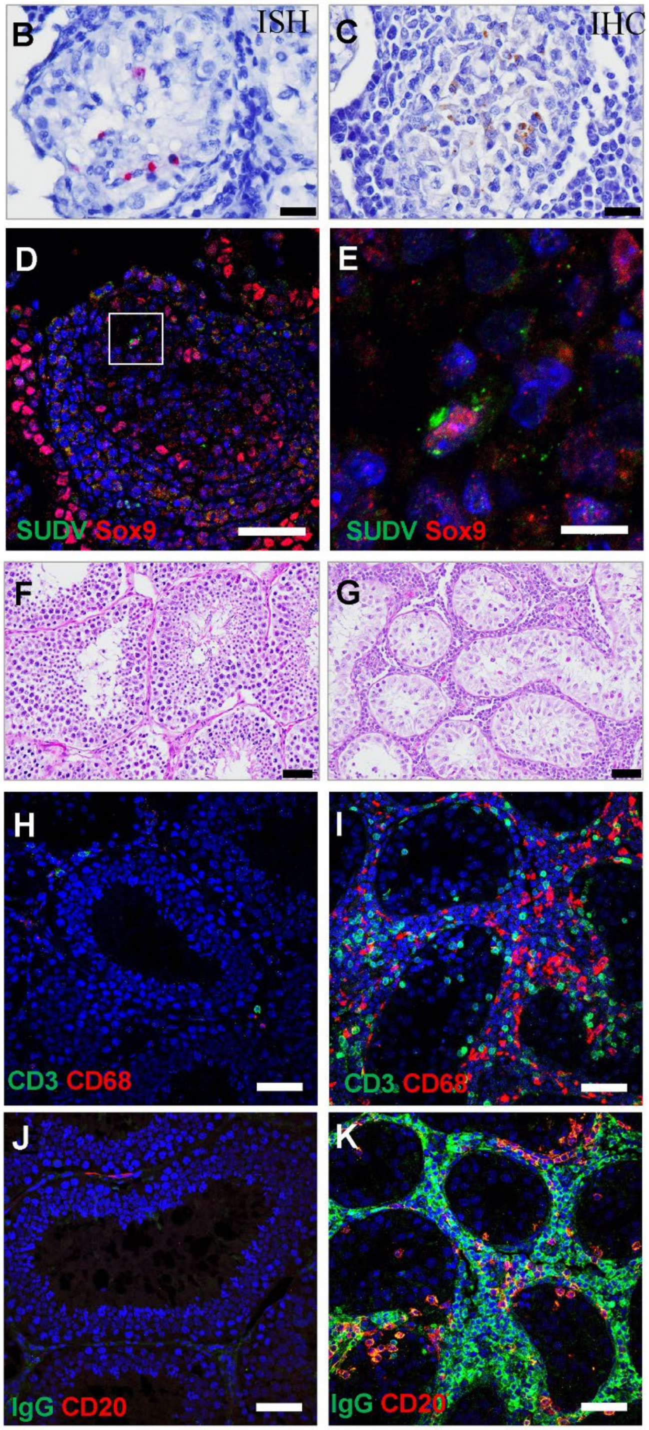

Appendix Figure 3. Testicular Sudan virus A) persistence in 1 (9%) and orchitis in 1 (9%) of 11 nonhuman primate (NHP) survivors (of after experimental acute infection without therapeutic intervention. B–C) SUDV genomic RNA (by RNA in situ hybridization, red in panel B) and antigen (by

immunohistochemistry, brown in panel C), were detected in the seminiferous tubules of testicular tissues of survivor 13. D–E) Immunofluorescence staining demonstrates SUDV antigen (green) detected in $Sox9^+$ Sertoli cells (red). E) Higher magnification of the white square inset in panel D. F–G) Testicular tissue sections of an uninfected control NHP (F) and survivor 13 stained with hematoxylin and eosin (H&E). H–K) Immunofluorescence staining demonstrating infiltration of $CD3^+$ T cells (green in H and I), $CD68^+$ macrophages (red in H and I), and $CD20^+$ B cells (red in J and K) and accumulation of antibodies (IgG, green in J and K) in the testicular tissues of survivor 13 (I and K) compared with the testicular tissues of uninfected controls (H and J). Nuclei were counterstained blue with 4′,6-diamidino-2-phenylindole. Scale bars indicate 20 μm (B–C), 10 μm (E), and 50 μm (D, F–K).

References

1. Zumbrun EE, Bloomfield HA, Dye JM, Hunter TC, Dabisch PA, Garza NL, et al. A characterization of aerosolized Sudan virus infection in African green monkeys, cynomolgus macaques, and rhesus macaques. Viruses. 2012;4:2115–36. PubMed https://doi.org/10.3390/v4102115
